# Robotics and Independent Living: Insights from Older Adults with Cognitive Impairment and Their Caregivers

**DOI:** 10.1002/alz70858_102316

**Published:** 2025-12-24

**Authors:** Delaram Sirizi, Morteza Sabet, Juanita‐Dawne Bacsu, Matthew Lee Smith, Elham Hariri Kashani, Zahra Rahemi

**Affiliations:** ^1^ Clemson University, Clemson, SC, USA; ^2^ Clemson University, Greenville, SC, USA; ^3^ University of Saskatchewan, Saskatoon, SK, Canada; ^4^ Texas A&M University, College Station, TX, USA; ^5^ Kashan University of Medical Sciences, Kashan, Isfahan, Iran (Islamic Republic of)

## Abstract

**Background:**

The prevalence of Alzheimer's disease and other cognitive disorders has been steadily rising among older adults, driven by aging populations and increased life expectancy worldwide. Declines in older adults’ cognitive and physical health pose challenges to maintaining their independence and aging in place. Robots can improve independent living and facilitate aging‐in‐place for people with cognitive impairment and Alzheimer's Disease. Despite recent innovations in healthcare robotics, their adoption among older adults, particularly those with progressive cognitive impairments, remains limited. This review examines perceptions about robots for independent living among older adults with cognitive impairment, their informal caregivers, and healthcare providers.

**Method:**

Five databases were systematically searched to identify qualitative and quantitative studies meeting the inclusion criteria. Content analysis was conducted to summarize the findings from the reviewed studies and a convergent parallel analysis was applied to integrate and interpret the findings comprehensively. Out of an initial pool of 348 studies, 16 met the inclusion criteria and were selected for the final review based on their alignment with the study's purpose and criteria.

**Result:**

From the review of the studies, qualitative themes are categorized into three main domains: user perceptions and experiences, barriers to adoption, and improvement suggestions. Quantitative findings highlight aspects such as usability, usefulness, acceptance, satisfaction, preferences, and barriers. Participants generally found the robots enjoyable and engaging to use; however, they emphasized the importance of adaptability, suggesting that the robots should be designed to accommodate the progressive decline in users’ cognitive abilities, ensuring continued relevance and usability over time.

**Conclusion:**

Our findings shed light on the dynamics of human‐robot interactions among older adults with cognitive impairments, emphasizing their potential to support independent living. These insights provide valuable guidance for developers, enabling them to design robots that better align with the needs, preferences, and abilities of this population. By enhancing user experiences and addressing the specific challenges of cognitive decline, these results can inform the development of adaptable and user‐friendly robots, ultimately improving their adoption and effectiveness in promoting aging‐in‐place.

